# Supplementary material for: Public misconceptions about dyslexia: The role of intuitive psychology
Source: PLoS One. 2021 Dec 2;16(12):e0259019. doi: 10.1371/journal.pone.0259019 (PMC8638985; doi:10.1371/journal.pone.0259019)
Supplement: S1 File — (DOCX) [file pone.0259019.s001.docx]

**Public misconceptions about dyslexia: the role of intuitive psychology**

**Supplementary Materials**

Iris Berent*^1^*

[i.berent@neu.edu](mailto:i.berent@neu.edu)

Melanie Platt*^1^*

[me.platt@northeastern.edu](mailto:me.platt@northeastern.edu)

^1^Department of Psychology

Northeastern University

**Additional Methods and Results**

Table S1

Table S2

Table S3

Table S4

Appendix S1

Appendix S2

Appendix S3

**Experiment 1**

**Results**

*A comparison to the scale’s midpoint.* To further evaluate the severity of each symptom, we also compare each mean against the scale’s “neutral” midpoint using a single sample t-test. In so doing, we sought to shed light on the *absolute* perceived likelihood of the symptom (as opposed to the comparison of visual and phonological symptoms to each other). The statistical results are presented in Table 1.

For most questions, responses differed significantly from the scale’s midpoint. Thus, people thought that John and Jack’s symptoms are indicative of a disorder, severe, they result from biological, but not attitudes and environmental causes, they likely manifest in their brain, run in the families, and likely are transferable to a clone, and this was further the case for both visual and decoding difficulties. But when asked about the person’s prognosis, here, people thought that decoding difficulties are likely to improve, but this was not the case for visual symptoms.

Table S1. The comparison of the mean ratings against the scale’s “neutral” midpoint.

| **Question** | **Visual difficulties** | | | **Decoding difficulties** | | |
| --- | --- | --- | --- | --- | --- | --- |
|  | *t(39)* | *p* | *d* | *t(39)* | *p* | *d* |
| 1. Disorder | 16.00 | **<0.0001** | 2.53 | 9.04 | **<0.0001** | 1.43 |
| 1. Severity | 7.38 | **<0.0001** | 1.17 | 3.39 | **<0.002** | 0.54 |
| 1. Environmental causes | -6.02 | **<0.0001** | -0.95 | -5.27 | **<0.0001** | -0.83 |
| 1. Biological causes | 9.86 | **<0.0001** | 1.56 | 6.43 | **<0.0001** | 1.02 |
| 1. Prognosis | 1.13 | 0.26 | 0.18 | 2.28 | **0.03** | 0.36 |
| 1. Affects brain | 13.68 | **<0.0001** | 2.16 | 7.95 | **<0.0001** | 1.26 |
| 1. In family | 11.82 | **<0.0001** | 1.87 | 6.92 | **<0.0001** | 1.09 |
| 1. In clone | 9.46 | **<0.0001** | 1.50 | 4.95 | **<0.0001** | 0.78 |

**Experiment 2**

Table S2. Statistical results in Experiment 2.

| **Question** | **Effect** | **F (1,39)** | **p** | **η**^2^_p_ |
| --- | --- | --- | --- | --- |
| 1. Severity | **Symptoms** | **4.22** | **0.05** | **0.10** |
|  | Test Type | 2.67 | 0.11 | 0.06 |
|  | Symptoms x Test Type | 1.09 | 0.30 | 0.03 |
| 2. Environmental causes | Symptoms | 0.58 | 0.45 | 0.01 |
|  | **Test Type** | **5.68** | **0.02** | **0.13** |
|  | Symptoms x Test Type | 1.17 | 0.29 | 0.03 |
| 3. Biological causes | Symptoms | 0.20 | 0.66 | 0.01 |
|  | **Test Type** | **10.68** | **0.00** | **0.21** |
|  | Symptoms x Test Type | 2.91 | 0.10 | 0.07 |
| 4. Prognosis | Symptoms | 1.14 | 0.29 | 0.03 |
|  | **Test Type** | **8.49** | **0.01** | **0.18** |
|  | Symptoms x Test Type | 0.06 | 0.81 | 0.00 |
| 5. Affects brain | Symptoms | 0.17 | 0.68 | 0.00 |
|  | *Test Type* | *3.58* | *0.07* | *0.08* |
|  | Symptoms x Test Type | 0.17 | 0.68 | 0.00 |
| 6. In family | Symptoms | 0.03 | 0.86 | 0.00 |
|  | Test Type | 0.93 | 0.34 | 0.02 |
|  | Symptoms x Test Type | 0.43 | 0.52 | 0.01 |
| 7. In Clone | Symptoms | 0.00 | 0.95 | 0.00 |
|  | **Test Type** | **8.30** | **0.01** | **0.18** |
|  | Symptoms x Test Type | 0.28 | 0.60 | 0.01 |

**Experiment 3**

Table S3. Statistical results contrasting participants in the Dualist and Physicalist groups in Experiment 3.

| **Question** | **Effect** | **F (1, 118)** | **p** | **η**^2^_p_ |
| --- | --- | --- | --- | --- |
| 1. Disorder | Priming | 0.17 | 0.68 | 0.00 |
|  | **Symptoms** | **36.74** | **<0.0001** | **0.24** |
|  | Priming x Symptoms | 0.00 | 0.96 | 0.00 |
| 2. Severity | Priming | 1.06 | 0.30 | 0.01 |
|  | Symptoms | 2.70 | 0.10 | 0.02 |
|  | **Priming x Symptoms** | **3.94** | **0.05** | **0.03** |
| 3. Environmental causes | Priming | 0.01 | 0.91 | 0.00 |
|  | **Symptoms** | **17.67** | **<0.0001** | **0.13** |
|  | Priming x Symptoms | 0.00 | 0.95 | 0.00 |
| 4. Biological causes | Priming | 0.12 | 0.73 | 0.00 |
|  | **Symptoms** | **7.54** | **0.01** | **0.06** |
|  | Priming x Symptoms | 0.02 | 0.89 | 0.00 |
| 5. Prognosis | Priming | 1.12 | 0.29 | 0.01 |
|  | **Symptoms** | **6.48** | **0.01** | **0.05** |
|  | Priming x Symptoms | 0.86 | 0.36 | 0.01 |
| 6. Affects brain | Priming | 1.18 | 0.28 | 0.01 |
|  | **Symptoms** | **4.71** | **0.03** | **0.04** |
|  | Priming x Symptoms | 0.90 | 0.34 | 0.01 |
| 7. In family | Priming | 0.05 | 0.83 | 0.00 |
|  | **Symptoms** | **10.10** | **<0.002** | **0.08** |
|  | Priming x Symptoms | 0.77 | 0.38 | 0.01 |
| 8. In Clone | Priming | 1.25 | 0.27 | 0.01 |
|  | **Symptoms** | **7.01** | **0.01** | **0.06** |
|  | Priming x Symptoms | 0.13 | 0.72 | 0.00 |

Table S4. Statistical results contrasting individual participants defined as “Dualists” and “Physicalists” in Experiment 3.

| **Question** | **Effect** | **F (1, 78)** | **p** | **η**^2^_p_ |
| --- | --- | --- | --- | --- |
| 1. Disorder | Group | 1.01 | 0.32 | 0.01 |
|  | **Symptoms** | **17.66** | **<0.0007** | **0.18** |
|  | Group x Symptoms | 0.00 | 0.97 | 0.00 |
| 2. Severity | Group | 1.68 | 0.20 | 0.02 |
|  | Symptoms | 0.01 | 0.91 | 0.00 |
|  | Group x Symptoms | 0.13 | 0.72 | 0.00 |
| 3. Environmental causes | Group | 1.25 | 0.27 | 0.02 |
|  | **Symptoms** | **18.93** | **<0.0004** | **0.20** |
|  | Group x Symptoms | 2.35 | 0.13 | 0.03 |
| 4. Biological causes | Group | 1.64 | 0.20 | 0.02 |
|  | *Symptoms* | *3.79* | *0.06* | *0.05* |
|  | Group x Symptoms | 0.11 | 0.74 | 0.00 |
| 5. Prognosis | Group | 0.16 | 0.69 | 0.00 |
|  | **Symptoms** | **3.99** | **0.05** | **0.05** |
|  | Group x Symptoms | 0.08 | 0.78 | 0.00 |
| 6. Affects brain | *Group* | *3.49* | *0.07* | *0.04* |
|  | *Symptoms* | *3.62* | *0.06* | *0.04* |
|  | Group x Symptoms | 1.09 | 0.30 | 0.01 |
| 7. In family | Group | 0.75 | 0.39 | 0.01 |
|  | **Symptoms** | **4.66** | **0.03** | **0.06** |
|  | Group x Symptoms | 0.18 | 0.67 | 0.00 |
| 8. In Clone | **Group** | **9.00** | **<0.004** | **0.10** |
|  | **Symptoms** | **8.14** | **0.01** | **0.09** |
|  | Group x Symptoms | 1.51 | 0.22 | 0.02 |

**Appendix S1: Materials in Experiment 1**

In this experiment, we ask you to reason about two people, John and Jack, who each suffer from difficulties with reading. We describe each person in a separate vignette. Please read each vignette carefully and then express your opinion about the case. Thank you!

* Note that the order of the decoding and visual vignettes was counterbalanced.

**Decoding**

John suffers from difficulties with reading. John can read text, but his reading is slow and deliberate. Moreover, when John is presented with new words, his difficulties are quite clear.


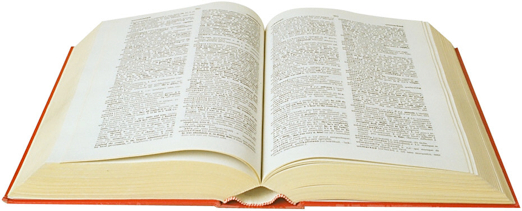


For example, when a typical reader is presented with the novel word *kat,* he immediately recognizes that this word sounds like the name of an animal. John does not. For him, *kat* is no more similar to an animal name than *vat*, for instance.

With this information in mind, consider various questions about the source of John’s difficulties.

1. Do you think it is likely that John’s symptoms are indicative of a reading disorder? (1= Very Unlikely, 4=As Likely as Not, 7=Very Likely)
2. How severe are John’s symptoms? (1= Very minimal; 4=neutral; 7=very Severe)
3. What is the source of John’s difficulty? Does it result from John’s own actions, attitudes, and life experiences? (1=strongly disagree; 4=neither agree nor disagree; 7=strongly agree)
4. Does it originate from his biological makeup? (1=strongly disagree; 4=neither agree nor disagree; 7=strongly agree)
5. How likely is it that John could improve his symptoms himself (e.g., by increasing his attention, trying harder, etc.)? (1= Very Unlikely, 4=As Likely as Not, 7=Very Likely)
6. How likely is it that the difficulty affects John’s brain? (1= Very Unlikely, 4=As Likely as Not, 7=Very Likely)
7. How likely is it that the difficulty runs in John’s family? (1= Very Unlikely, 4=As Likely as Not, 7=Very Likely)
8. Consider the following hypothetical scenario. Suppose it were possible to clone a baby out of John’s DNA. The clone would entirely replicate John’s genes, but it would be raised in an entirely different family and environment. Once this infant matures, how likely is it to exhibit the same difficulties as John? (1= Very Unlikely, 4=As Likely as Not, 7=Very Likely)

**Visual**

Jack has difficulties with another aspect of reading. His difficulties concern the visual identification of letters. Like John, Jack can read, albeit slowly and with great effort. But when Jack is presented with printed words very briefly, his difficulties become clear.


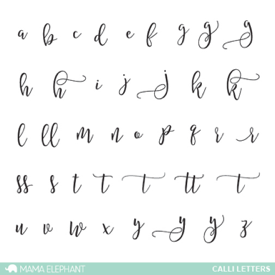


For example, when typical readers see the letters *b* and *d* they can readily tell them apart, even when presented for only a brief moment. Jack, however, fails to discriminate between these letters. When letters are presented, he treats *b* and *d* as if they were the same.

With this information in mind, consider various questions about the source of Jack’s difficulties.

1. Do you think it is likely that Jack’s symptoms are indicative of reading disorder? (1= Very Unlikely, 4=As Likely as Not, 7=Very Likely)
2. How severe are Jack’s symptoms? (1= Very minimal; 4=neutral; 7=very Severe)
3. What is the source of Jack’s difficulty? Does it result from Jack’s own actions, attitudes and life experiences? (1=strongly disagree; 4=neither agree nor disagree; 7=strongly agree)
4. Does it originate from his biological makeup? (1=strongly disagree; 4=neither agree nor disagree; 7=strongly agree)
5. How likely is it that Jack could improve his symptoms himself (e.g., by increasing his attention, trying harder, etc.)? (1= Very Unlikely, 4=As Likely as Not, 7=Very Likely)
6. How likely is it that the difficulty affects Jack’s brain? (1= Very Unlikely, 4=As Likely as Not, 7=Very Likely)
7. How likely is it that the difficulty runs in Jack’s family? (1= Very Unlikely, 4=As Likely as Not, 7=Very Likely)
8. Consider the following hypothetical scenario. Suppose it were possible to clone a baby out of Jack’s DNA. The clone would entirely replicate Jack’s genes, but it would be raised in an entirely different family and environment. Once this infant matures, how likely is it to exhibit the same difficulties as Jack? (1= Very Unlikely, 4=As Likely as Not, 7=Very Likely)

**Appendix S2: Materials in Experiment 2:**

In this experiment, we ask you to reason about two pairs of twins, who each suffer from difficulties with reading. We describe the disorder of each case in a separate vignette. Please read each vignette carefully and then express your opinion about the case. Thank you!

*Note that within each vignette, the order for the brain/behavioral test questions was counterbalanced; presented below is the brain-behavior order. The decoding and visual vignette order was also counterbalanced.

**Decoding**

John and Jack are identical twins, who suffer from difficulties with reading. They can both read text, but their reading is slow and deliberate. Moreover, when presented with new words, their difficulties are quite clear. For example, when a typical reader is presented with the novel word *kat,* he immediately recognizes that this word sounds like the name of an animal. But John and Jack do not. For them, *kat* is no more similar to an animal name than *vat*, for instance.


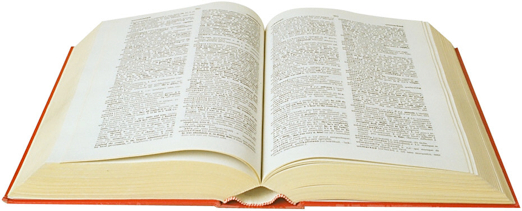


John and Jack have a friend who is a reading specialist, and she urges them to be screened for reading disorders. She told them that research has shown that people read by instinctively using abstract rules the match letters with sounds. People typically deploy these rules automatically, without conscious awareness or formal training. But when these rules are impaired, this can lead to a reading disorder. The friend is worried that John and Jack each suffers from a reading disorder that disrupts these rules.

John and Jack decide to heed her advice, and they schedule a reading assessment by a reading disorder clinician. During the assessment, they each see novel words, and their responses are assessed. John’s performance is evaluated by scanning his brain, whereas for Jack, the clinician assesses reading speed behaviorally.

Both tests are strictly normed, so the clinician determined that John and Jack’s performance was abnormal. But for John, this conclusion was based on a brain test whereas for Jack, the results came from a behavioral experiment.

With this information in mind, consider various questions about the source of John’s condition (diagnosed by the ***brain*** test).

1. How severe is John’s condition? (1= Very minimal; 4=neutral; 7=very Severe)
2. What is the source of John’s condition? Does it result from John’s own actions, attitudes, and life experiences? (1=strongly disagree; 4=neither agree nor disagree; 7=strongly agree)
3. Does it originate from his biological makeup? (1=strongly disagree; 4=neither agree nor disagree; 7=strongly agree)
4. How likely is it that John could improve his symptoms himself (e.g., by increasing his attention, trying harder, etc.)? (1= Very Unlikely, 4=As Likely as Not, 7=Very Likely)
5. How likely is it that the condition affects John’s brain? 1= Very Unlikely, 4=As Likely as Not, 7=Very Likely)
6. How likely is it that the condition runs in John’s family? (1= Very Unlikely, 4=As Likely as Not, 7=Very Likely)
7. Consider the following hypothetical scenario. Suppose it were possible to clone a baby out of John’s DNA. The clone would entirely replicate John’s genes, but it would be raised in an entirely different family and environment. Once this infant matures, how likely is it to exhibit the same language condition as John? (1= Very Unlikely, 4=As Likely as Not, 7=Very Likely)

Now, consider the results of Jack’s evaluation, whose condition was diagnosed using the ***behavioral*** test.

1. How severe is Jack’s condition? (1= Very minimal; 4=neutral; 7=very Severe)
2. What is the source of Jack’s condition? Does it result from Jack’s own actions, attitudes, and life experiences? (1=strongly disagree; 4=neither agree nor disagree; 7=strongly agree)
3. Does it originate from his biological makeup? (1=strongly disagree; 4=neither agree nor disagree; 7=strongly agree)
4. How likely is it that Jack could improve his symptoms himself (e.g., by increasing his attention, trying harder, etc.)? (1= Very Unlikely, 4=As Likely as Not, 7=Very Likely)
5. How likely is it that the condition affects Jack’s brain? 1= Very Unlikely, 4=As Likely as Not, 7=Very Likely)
6. How likely is it that the condition runs in Jack’s family? (1= Very Unlikely, 4=As Likely as Not, 7=Very Likely)
7. Consider the following hypothetical scenario. Suppose it were possible to clone a baby out of Jack’s DNA. The clone would entirely replicate Jack’s genes, but it would be raised in an entirely different family and environment. Once this infant matures, how likely is it to exhibit the same language condition as Jack? (1= Very Unlikely, 4=As Likely as Not, 7=Very Likely)

**Visual**

Like John and Jack, Paul and Greg are also identical twins who suffer from reading difficulties, but their challenges arise from a different source .

Paul and Greg’s difficulties concern the visual identification of letters. Like John and Jack, Paul and Greg can read, albeit slowly and with great effort. But when Paul and Greg are presented with printed words very briefly, their difficulties become clear. For example, when typical readers see the letters *b* and *p*, they can readily tell them apart, even when presented for only a brief moment. Paul and Greg, however, fail to discriminate between these letters. When letters are presented, they both treat *b* and *p* as if they were the same.


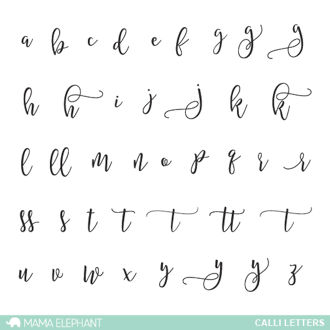


Paul and Greg recently ran into a friend who practices as a reading specialist. Paul told the friend of their difficulties and the friend, worried, suggested that the brothers be assessed for a reading disorder. She told Paul that when people learn to read, they develop the ability to discriminate between mirror-symmetrical letters, such as *b* and *d.* The friend worries that Paul and Greg might have a disorder that impairs this visual process.

The brothers heed the friend’s advice, and obtain an assessment for their condition. During the assessment, both brothers are presented with brief displays including similar letters, such as *b* and *d*, and their responses are assessed. Paul’s performance is evaluated by scanning his brain activity, whereas for Greg, the clinician assesses reading speed behaviorally.

Both tests are strictly normed, so the clinician determined that Paul and Greg’s performance was abnormal. But for Paul, this conclusion was based on a brain test whereas for Greg the results came from a behavioral experiment.

With this information in mind, consider various questions about the source of Paul’s condition (diagnosed by the ***brain*** test).

1. How severe is Paul’s condition? (1= Very minimal; 4=neutral; 7=very Severe)
2. What is the source of Paul’s condition? Does it result from Paul’s own actions, attitudes, and life experiences? (1=strongly disagree; 4=neither agree nor disagree; 7=strongly agree)
3. Does it originate from his biological makeup? (1=strongly disagree; 4=neither agree nor disagree; 7=strongly agree)
4. How likely is it that Paul could improve his symptoms himself (e.g., by increasing his attention, trying harder, etc.)? (1= Very Unlikely, 4=As Likely as Not, 7=Very Likely)
5. How likely is it that the condition affects Paul’s brain? 1= Very Unlikely, 4=As Likely as Not, 7=Very Likely)
6. How likely is it that the condition runs in Paul’s family? (1= Very Unlikely, 4=As Likely as Not, 7=Very Likely)
7. Consider the following hypothetical scenario. Suppose it were possible to clone a baby out of Paul’s DNA. The clone would entirely replicate Paul’s genes, but it would be raised in an entirely different family and environment. Once this infant matures, how likely is it to exhibit the same language condition as Paul? (1= Very Unlikely, 4=As Likely as Not, 7=Very Likely)

Now, consider the results of Greg, whose condition is diagnosed using the ***behavioral*** test.

1. How severe is Greg’s condition? (1= Very minimal; 4=neutral; 7=very Severe)
2. What is the source of Greg’s condition? Does it result from Greg’s own actions, attitudes, and life experiences? (1=strongly disagree; 4=neither agree nor disagree; 7=strongly agree)
3. Does it originate from his biological makeup? (1=strongly disagree; 4=neither agree nor disagree; 7=strongly agree)
4. How likely is it that Greg could improve his symptoms himself (e.g., by increasing his attention, trying harder, etc.)? (1= Very Unlikely, 4=As Likely as Not, 7=Very Likely)
5. How likely is it that the condition affects Greg’s brain? 1= Very Unlikely, 4=As Likely as Not, 7=Very Likely)
6. How likely is it that the condition runs in Greg’s family? (1= Very Unlikely, 4=As Likely as Not, 7=Very Likely)
7. Consider the following hypothetical scenario. Suppose it were possible to clone a baby out of Greg’s DNA. The clone would entirely replicate Greg’s genes, but it would be raised in an entirely different family and environment. Once this infant matures, how likely is it to exhibit the same language condition as Greg? (1= Very Unlikely, 4=As Likely as Not, 7=Very Likely)

**Appendix S 3: Materials in Experiment 3:**

Step 1: Vignettes

**Vignette – dualism priming condition:**

In accordance with theories of ancient Greek and early modern philosophers, many contemporary scientists believe that a person’s mind and body are two distinct entities.

This thesis, which is called "mind-body dualism", is based on the assumption that immaterial mental states are different from, though dependent on, physical brain states. Thus, unlike the brain, the mind refers to a state of consciousness not equated with physiology.

Thus, unlike the brain, the mind refers to a state of consciousness not equated with physiology.

This mind-body distinction has been proposed to account for the observation that mental phenomena appear to be qualitatively and substantially different from the physical bodies on which they appear to depend.

We do not perceive ourselves as “machines made of meat.” Instead, we feel as if we occupy our bodies. This is also suggested by our day-to-day experiences. It is quite common to distinguish between “my body” and “my self.”

For instance, our bodies may become injured or ill even though our minds are still active and alert. Our mental experience is also private, reinforcing the feeling that it is somehow separate.

Further, there is still no scientific consensus as to precisely how mental life emerges from a physical body, and many scientists doubt there will ever be a satisfying explanation.

Therefore, some scientists believe that even a computer that could perfectly mimic human beings, including humans’ emotional expressions (like joy, fear, anger, etc.), would never be able to actually experience them. Thus, this computer’s actions would be indistinguishable in every way from those of a normal human being, except that it lacks conscious experience or sentience.

In this same vein, we quite naturally acknowledge that identical twins are separate human beings who have their own individual conscious experiences, even though they are completely identical on a biological level.

In sum, the term “mind-body dualism” describes the proposition that a person’s mind and body are two distinct entities.

**Vignette – physicalism priming condition:**

In accordance with theories of modern philosophers, most contemporary scientists believe that a person’s mind and body are rooted in the same physical substances.

This thesis, which is called "physicalism", is based on the assumption that mental states are nothing but physical brain states. Consciousness and the mind can thus be described as properties of matter and as a function of a biological substance: the brain.

Physicalism has been proposed to account for the many recent research findings in the field of neuroscience, which show that any mental activity is reflected in brain activity. Minds are what brains do.

We readily accept that apparently solid objects are actually mostly empty space, consisting of tiny particles and fields of energy. Consistently, modern scientists view the mind as emerging from the forces of living matter, or as a mere epiphenomenon of this matter.

This is also suggested by our day-to-day experiences. For instance, we know that physical pain or a decreased blood glucose level can affect our mood, and that psychoactive drugs can rapidly change our perception and our behavior.

Further, as there is growing scientific consensus as to how mental life emerges from a physical body, most modern scientists do not doubt the neural basis of thought, i.e., the physical origin of mental life.

Therefore, some scientists believe that a computer that could perfectly mimic human beings, including humans’ emotional expressions (like joy, fear, anger, etc.), would be able to actually experience them.

Thus, this computer’s actions would be indistinguishable in every way from those of a normal human being, and it would experience consciousness and sentience.

In this same vein, we quite naturally acknowledge that identical twins often share personality symptoms, as they are completely identical on a biological level.

In sum, the term “physicalism” describes the proposition that a person’s mind and body are both rooted in the same physical substances.

Step 2: Manipulation check:


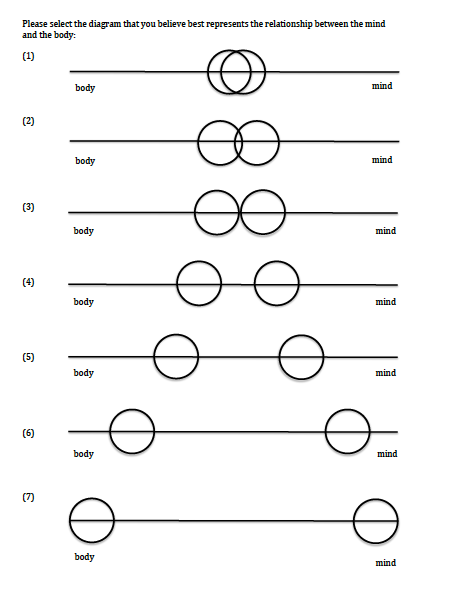


Step 3: Same decoding/visual vignettes as in Experiment 1
