# Supplementary material for: Public misconceptions about dyslexia: The role of intuitive psychology
Source: PLoS One. 2021 Dec 2;16(12):e0259019. doi: 10.1371/journal.pone.0259019 (PMC8638985; doi:10.1371/journal.pone.0259019)
Supplement: Attachment — Submitted filename: reply to reviewers.docx [file pone.0259019.s003.docx]

**Reviewer #1**

This paper presents three studies that explore how the symptoms associated with dyslexia influence people’s perceptions of the condition. In Experiment 1, the authors examined how telling participants that a person is experiencing visual or decoding difficulties influences their beliefs about the severity and biological basis of the illness. In Experiment 2, they examined how the symptoms interact with whether the illness was diagnosed through a behavioral or brain test. In Experiment 3, they explore how the symptoms interact with people’s dualistic beliefs. The idea of dualistic beliefs influencing people’s reasoning about dyslexia is interesting, however many aspects of the paper need considerable revisions.

Major issues
1. The authors should consider expanding and reorganizing the introduction. Currently the introduction reads somewhat disjointed, with each paragraph presenting new information, but little connecting paragraphs or articulating the argument more explicitly for the reader. Additionally, the authors used jargon terms in the introduction without defining or explaining them. Some examples include “deep orthography,” “intuitive psychology,” “intuitive biases,” “dualism,” and “essentialism.” Explaining these terms would help a naïve reader understand the introduction. I also found it interesting that the introduction does not review literature on laypeople’s views on mental illness. The authors cite some of this work in the discussion, but some mention of how people reason about mental illness might be useful in the introduction. The authors should consider moving some of the information presented in Part 2 and the general discussion to the introduction as these sections articulate their argument more and do a good job at summarizing prior research.

Let me start by thanking you for your careful read and the extremely helpful comments. In preparing the previous draft, we were struggling with how to speak to the multiple potential audiences of this piece—the reading specialists, who have little background in intuitive psychology, and the readership who is interested in intuitive psychology (but lacks expertise in reading science). In the previous manuscript, we thus opted for a data-driven approach, with minimal discussion of theory. Evidently, this rendered our investigation pretty opaque to both groups.

Reading your comments, we now see where, precisely, the ambiguities lie. In light of these comments, we have massively revised the Introduction, explaining how our investigation speaks to two distinct literatures—the one on intuitive psychology generally, and attitudes towards mental disorders, specifically; the other is the literature on dyslexia, and laypeople’s attitudes towards it. We have also clarified these various terms, so that this paper can speak to these two different groups of researchers.

2. One aspect that was not clear to me as a reader was the intended audience for this paper. If the audience is cognitive scientist interested in people’s concepts, then the authors should consider justifying why examining people’s views of dyslexia (specifically) might be useful. If the audience is dyslexia researchers, then more information on the discussion about how these biases might influence issues such as intervention choices or stigma might be needed. If they want the article to appeal to both groups, the authors should consider addressing both issues.

We have now followed your helpful suggestion. As noted, this research carries both translational implications to dyslexia remediation as well as foundational implications for cognitive science interested in intuitive reasoning, and as such, we hope to appeal to both groups. We have revised the paper accordingly.

3. The authors should consider explaining how their review of the literature leads to their hypotheses. When I first read the hypothesis for Experiment 1 “biological disorders are more likely to affect sensory capacities than cognitive learning mechanisms” (pg. 4) I was not sure how that was related to the other issues discussed in the introduction. My guess is that this hypothesis is related to dualism, but this concept has not been introduced until a later paragraph.

We now review these multiple literatures in the Introduction. We first examine the scientific literature on dyslexia and contrast it with laypeople’s attitudes on the topic; we next move to consider how people reason about the (typical psyche), and the role of Dualism and Essentialism in shaping these attitudes. We then move to consider how these principles have been invoked in the past literature on disorders, specifically, psychiatric disorders. Finally, we articulate a novel proposal as to how the same framework we had previously explain laypeople’s psychological reasoning (about the typical psyche and mental disorders) can capture laypeople’s attitudes towards dyslexia. In light of this theoretical Introduction, it should now be clear why our experiments choose these particular manipulations.

4. I found the sample sizes in all the studies to be fairly small, particularly for researchers using online recruitment methods such as Mechanical Turk. In addition, the authors do not present a power analysis or a justification for their sample size. Even though Experiment 1 has only 40 participants, it has the benefit that Experiment 3 uses similar methods with a higher sample size (120 participants), effectively serving as a replication. However, there is no replication of Experiment 2, which also has only 40 participants, but was additionally testing for interactions. Given that there is no justification for the sample size of any of the studies, I have doubts on whether these studies were adequately powered to detect interactions. For example, some of the effects that were not significant in Experiment 1 were significant in Experiment 3 (which had a higher sample size). It would be useful to have a high-powered replication of the effects shown on this paper.

Because no previous research has examined these particular manipulations in dyslexia, we have based our estimates of sample size on our previous research, in which we asked people to reason about the embodiment and innateness of sensory and epistemic states (Berent, Platt, & Sandoboe, 2019; Berent, Platt, & Sandoboe, in press) as well as our related work on psychiatric disorders (Berent & Platt, 2021) and the previous priming study by Forstmann and colleagues (Forstmann, Burgmer, & Mussweiler, 2012).

We recognize that these sample sizes are smaller than in some of the other literature. As noted, however, our studies took steps to eliminate inattentive responses (by requiring all participants to provide a justification of their answer); this is now described as part of the Methods section. As explained below, we have also noticed an error in the statistical analysis of Experiment 1—several results that were previously reported as non-significant are now significant.

5. There should be more information about the actual materials and procedures of the study in the body of the manuscript. Currently, the actual stimuli are in the supplemental materials, but the authors don’t present a description of their methods in the Method section. Additionally, please make it clear in the description of study 2 that both of the twins in one vignette had either visual or decoding symptoms.

Thanks for these suggestions—the Methods sections have been revised to describe the materials; we also note that the two twins (in Experiment 2) showed the same type of difficulty (either visual or phonological).

6. More information is needed as to what the author’s hypotheses were and how these hypotheses relate to their outcome measures. For example, one hypothesis presented is that visual disorders would be perceived as more biological. This hypothesis explains why the authors asked about environmental and biological causes, whether the disorder affects the brain, runs in the family, and a clone would have it. However, it is less clear why some of the other questions (e.g., severity, prognosis, indicative of a reading disorder) are being asked. A revised introduction could explain why these variables are being included, how they relate to their overall argument, and what the authors hypothesized (conversely, if these variables are exploratory, they should be labelled as such).

Thank you for this helpful suggestion. Now that the theoretical background is presented in the Introduction, it is easier for us to motivate our predictions a priori, and spell them out. We have now done so for all experiments.

7. In page 7, the authors claim, “These results make it clear that not only are laypeople more likely to associate dyslexia with visual difficulties (rather than decoding difficulties), but that their attitudes towards the symptoms of the disorder and its etiology are linked.” The second part of the statement is support by their data as people who read the disorder was associated with visual impairments thought it was more likely for it to have biological causes. However, it is not clear to me which aspect of their data speaks to the first part of their statement. The authors never asked participants whether dyslexia was associated with visual or decoding difficulties. They did have a question asking whether the symptoms are indicative of a reading disorder (generally), not dyslexia (specifically). Given some of the work the authors review in the introduction, I can see why they make this claim, however, the link between the claim and the data they present needs to be articulated further. This is particularly important as this claim is the motivation for study 2.

Here, we assume that people interpret “dyslexia” as synonymous with “reading disorder”. We are unaware of any evidence that laypeople link developmental reading disorders with any other clinical labels. In this regard, dyslexia differs from major psychiatric disorders. Laypeople do not automatically link “psychiatric disorders” with any particular illness (e.g., schizophrenia); but for developmental reading disorders, dyslexia is the only viable category. For the sake of accuracy, however, we have now revised the text to refer to “reading disorder”, specifically.

8. In page 15 the authors decide to split the sample based on their responses to the dualism question. I have several methodological concerns about this analysis. First, it appears to me that this analysis is exploratory, and it would be useful if the authors labelled it as such. However, if this analysis is not exploratory, then it needs to be motivated in the hypotheses section. Second, the authors exclude participants whose attitude did not align with their experimental condition (e.g., they eliminated participants that rated below the midpoint only if they were in the dualist condition). Their reasoning for doing this was not explained. This decision is concerning because, as shown by the means in page 13, the averages for both conditions are below the midpoint. This creates an imbalance in the conditions where almost all the participants in the physicalist condition are kept in the analysis, but only one third of those in the dualist condition are kept in the analysis. Third, the methodological literature suggests that dichotomizing continuous variables often leads to lower power and higher spurious findings (see MacCallum, 2002). Rather than dichotomizing their predictors, the authors could examine the interaction between mind-body distance (as a continuous variable) and symptom condition in a regression.

We agree that this analysis is exploratory, and we have thus decided to remove it from the paper. We believe that the results of the main experiments are sufficient to support our claims.

9. In page 17, the authors claim that “our results suggest that (a) people are more likely to view visual symptoms of dyslexia as indicative of a hereditary brain disorder.” Although the hereditary portion is found in their data, the “brain disorder” claim was only significant in Study 3 (the p-values for studies 1 and 2 are above .100). There are other instances in the manuscript where results that do not reach the cut-off value of .050 are describe in the discussion in a way that implies they are significant. The authors should consider qualifying all such statements describing how they were not significant, or only significant in one study.

Thank you for your careful read. In light of your comments, we went back and doubled-checked our statistical calculation. We found that our previous reports were erroneous (the t-tests were incorrectly computed for distinct, rather than for paired samples). The revised report shows that visual symptoms were considered as more likely to affect the brain in both Experiments 1 & 3; Experiment 2 further shows that symptoms that are diagnosed by a brain test are more likely to be viewed as innate (relative to ones diagnosed behaviorally). Additionally, all experiments show a significant correlation between the perceived likelihood of a disorder to affect the brain and its perceived innateness.

Minor issues
• Please report demographic information for the participants in the body of the text rather than in the supplemental materials. When I examined the supplemental materials, I found no information about participants’ age, gender and race or ethnic information. It would be useful to report this information. Additionally, the reporting of the demographic information in the supplemental materials combines all three studies (or at least that is my impression from reading it). Please report the demographic information separately for each study.

We now include the demographic information available to us for each experiment separately, within the body of the text. Unfortunately, this information only specifies the level of education—we did not collect information about gender, race or ethnicity.

• Please include how much money participants received for participating in the studies and how long it took them to complete the studies.

This is now indicated (in the Methods of Exp. 1).

• It is common to include attention checks in Mechanical Turk studies to make sure that participants are reading carefully. Did the authors include attention checks? Did all participants pass the checks?

As described in the revision, to be included in the sample, we required all participants to provide a coherent answer to a question, asking them to explain their rating. “Coherent” was defined liberally—we accepted any justification, as long as it was not copied verbatim from the text (which could be done by a bot). To obtain the desired sample (e.g., N=40), we thus ran a larger sample (of about 150% the desired size). We then inspected the justifications and eliminated participants who did not provide justification. The final sample corresponds to the first N participants who provided justification (e.g., the first 40 participants who provided a justification); “first” here refers to the order of their enrollment in the study. This inclusion was done prior to any inspection of the data, in a manner that is utterly blind to participants’ responses. This procedure was adopted in Experiment 1 and 3; due to an oversight on our part, we failed to include the comprehension question in Experiment 2, so this procedure was not applied therein. our part. We have now described this procedure in the Method of Experiment 1.

• Where there any order effects?

Thank you for this suggestion. We did not test for order effects, as we do not believe that they are directly relevant to our inquiry. In our opinion, order effects shed light on whether participants’ responses were influenced by the salience of our manipulations (e.g., whether the visual symptoms were presented first). While we have no doubt that salience could well matter, our question here is different: we ask whether the conditions of interest differ *even when order is controlled*. We are concerned that inspecting the effects of order will send us on a tangent, and ultimately, obscure our main questions and findings. For this reasoning, we did not pursue an analysis of order.

• Please present the full range of the y-axis in Figures 2 and 3 (this would match the range shown in Figures 1, 4 and 5)

All figures are now presented with the full scale; the only exception is Figure 3, where we prefer to maintain the current scale so as to render the effects of interest clearly visible. We now indicate that the axes were truncated.

• If at all possible, it would be nice if the same color coding for the conditions is used in all the graphs.

Done

• I would be useful to report the simple effects for the test by symptom interaction in the results section rather than in the discussion section (pg. 11).

We agree that, in general, it’s better to limit the report of the statistical results to the “result” section. In the present case, however, this particular statistical test is exploratory, as it is conducted to shed light on an unexpected finding. For this reason, we believe that it is appropriate to report it in the Discussion.

• Has the diagram scale to measure mind-body distance been used before? How should this measure be interpreted? It looks like from 4 onwards people are selecting that the mind and body do not overlap, does it matter how separate the circles are?

Our manipulation of Dualism (including the use of the circle diagram) follows the previous studies of (Berent et al., in press; Forstmann et al., 2012)—these sources are now cited. We now clarify (in the Methods) that high ratings indicate greater separation between the mind and the body.

• The authors describe in their introduction to part 2 that people are “inherent dualists,” however by looking at the means on their mind-body distance task, even the group primed to think in a dualistic manner had scores below the mid-point (suggesting they are more physicalist). How do the authors make sense of these conflicting findings?

We do not believe these results are in conflict. Unlike the scales used in our main manipulation (where 4 indicated “neutral”), in the circle task, the circles indicating the mind and body differ already for the scale rating of 1. Accordingly, the mean rating of 3.63 certainly indicates a difference. As we further note in the revision, responses in the Dualist condition (in Experiment 3) were similar to the ones in Experiment 1 (without priming)—it is the Physicalist condition (in Experiment 3) that is responsible for the difference. These results, then, support our contention that people are by default Dualists.

• The footnote on page 15 should be incorporated into the body of the text.

This footnote is no longer relevant (it formed part of an exploratory analysis of participants’ attitudes towards the mind-body link, which is now removed)

• There are inconsistencies in the manuscript as to how many significant digits are reported for the p-values (ranges from 2 to 4).

Our apologies. We have now checked this thoroughly.

• When conducting ANOVA’s please report whether they are between, repeated measures, or mixed.

Done

• Please report standard deviation every time you report means.

Done

• I ran the paper through statcheck.io and found three instances where the p-values were not consistent. Please double check you that all statistics are accurate.

We apologize for these errors. We have checked all results carefully.

• Why test each average against the mid-point of the scale? (I am not saying this is wrong, but rather that it needed some justification as it rarely came up in the results).

We now spell out the logic in the SM. As explained, the comparison against the midline helps gauge the *absolute* valence of participants attitudes (as opposed to the *relative* attitudes for visual vs. phonological symptoms).

• There are stylistic and typographical issues in the manuscript (how quotations are cited, missing parentheses, extra punctuation).

Our apologies—we have proofread the manuscript thoroughly.

**Reviewer #2**:

This study had two main aims: (1) examining the relationship between peoples’ attitudes towards the symptoms of dyslexia (related to visual or phonological decoding difficulties) and their views on etiology and severity; (2) examining the relationship between the perceived mind-body linkage and the view of heritability of dyslexia with an implication of Dualism and Essentialism. To address these aims, the researchers designed 3 experiments. The 1st experiment studied the link between people’s attitudes towards the symptoms of dyslexia and its perceived etiology. Forty participants were asked to read two vignettes, featuring two individuals with reading difficulties associated with different symptoms, one with visual difficulties and another with phonological decoding. Participants were asked to evaluate the likelihood of reading disorder, severity and etiology. Eight t-tests were run to examine if the evaluation of reading disorder differed between individuals presented with visual-related vs. decoding-related symptoms. Overall the results showed that people tended to view visual difficulties (vs. decoding difficulties) as more indicative of reading disorder, more severe, more likely to be biologically caused, and more heritable. The 2nd experiment examined if people’s attitudes towards the etiology of reading disorder were influenced by whether the disorder was diagnosed by brain vs. behavioral measures. Forty participants read two vignettes: (1) one individual was diagnosed with dyslexia using brian test; (2) another individual was diagnosed with dyslexia using behavioral results. Participants were then asked seven questions on the etiology of reading disorder for each individual. Seven 2 Symptoms (Decoding/Visual) x 2 Test (Brain/Behavior) ANOVAS were run. Overall the results showed that when a diagnosis was done by brain test (vs. behavioral test), participants considered dyslexia as more innate, less likely to be caused by environment , more likely linked to biological basis, had poorer prognosis, more likely to affect the brain, and more likely to transfer to genetic clone. The 3rd experiment aimed at (1) replicating the results in Experiment 1, and (2) examining whether people’s attitudes towards the disorder would vary when the perceived mind-body linkage was different. A total of 120 participants were divided into 2 groups: Dualism group read an essay that describes the mind as distinct from the body; and Physicalism group read an essay that describes the mind and body as the same. Reading these essays served as a priming paradigm. Then, the participants read two vignettes, one featuring an individual suffering from a reading difficulty with decoding difficulty, and another featuring an individual suffering from visual symptoms. Similar to Experiment 1, participants were asked to evaluate the likelihood of a disorder, its severity, and etiology. Overall, the results showed people’s attitudes towards the severity of the disorder were modulated by the priming conditions. Participants who were primed with Dualism content considered reading disorders associated with visual symptoms as more severe than decoding difficulties. However, no significant difference in severity was observed when participants were primed in the Physicalism condition.

Overall, this study is interesting and the results will give us insight into the general public’s attitudes towards the etiology of dyslexia and the underlying causes of misconceptions about the disorder. The experiments, especially the vengitetes in the 3 experiments were well designed. Though the experiments are sound, the statistical analyses and the interpretation appear to be problematic. The researchers should address these fundamental issues; otherwise, the results and interpretation would not stand.

(1) The authors interpreted results with p-value = 0.5 as if it was significant. Furthermore, all the analyses had multiple t-tests and ANOVAS but no corrections were applied. If a multiple correction were to apply in the analyses, all results in Experiment 1 would be null.

First, thank you for your careful read of our paper and the helpful comments.

We apologize for this oversight in statistical reporting—we have now carefully inspected all results to make sure they are described appropriately; we now refer to results with p=.05 as *marginally* significant.

Regarding corrections in Experiment 1: following the advice of Reviewer 1, we have now massively revised the Introduction to explain our predictions upfront. Now that the theoretical background is spelled out, I hope it becomes clear that the multiple tests performed in Experiment 1 are not a “fishing expedition”. Rather, these measures operationalize the two theoretical constructs of interest: embodiment and essentialism. ***Embodiment*** is gauged by the “affects the brain question”; the questions regarding biological origin, innateness (i.e., “runs in the family, “transfers to a genetic clone”, and their converse, “results from one’s experience”), immutability (severity and prognosis) are all known aspects of ***essentialist*** thinking. These two constructs—embodiment and essentialism—are based on a-priori planned hypothesis, and for this reason, we did not apply corrections.

(2) ANOVAS. The researchers did not mention what kind of anovas were used. Are they repeated measures? Clarification is needed

We have now clarified the design in all analyses. Thank you for this suggestion..

Reviewer #1: **Yes:** David Menendez

Reviewer #2: No

Berent, I., & Platt, M. (2021). Essentialist biases about psychiatric disorders: Brain disorders are presumed innate. *Cognitive Science, 45*(4), e12970. doi: 10.1111/cogs.12970

Berent, I., Platt, M., & Sandoboe, G. M. (2019). People’s intuitions about innateness. *Open Mind: Discoveries in CognitiveScience, 3*, 101-114.

Berent, I., Platt, M., & Sandoboe, G. M. (in press). Empiricism is natural: it arises from dualism and essentialism. *Oxford Studies in Experimental Philosophy*.

Forstmann, M., Burgmer, P., & Mussweiler, T. (2012). “The Mind Is Willing, but the Flesh Is Weak”: The Effects of Mind-Body Dualism on Health Behavior. *Psychological Science, 23*(10), 1239-1245. doi: 10.1177/0956797612442392
