# Supplementary material for: Public misconceptions about dyslexia: The role of intuitive psychology
Source: PLoS One. 2021 Dec 2;16(12):e0259019. doi: 10.1371/journal.pone.0259019 (PMC8638985; doi:10.1371/journal.pone.0259019)
Supplement: Attachment — Submitted filename: reply to reviewers.docx [file pone.0259019.s004.docx]

**Reviewer #1**: This is a very responsive revision. The authors have addressed all of the comments I made in my first review. The new introduction, although long, adequately situates the study and motives the hypotheses. The methods are now much clearer, and the results easier to read. All the statistics reported seem to be in order, so the authors did a good job at checking them.

We are delighted—thank you for the helpful comments.

I have only two minor comments.

1. The authors claimed in several sections that the people are more likely to “essentialize visual symptoms because they perceive them as more strongly anchored in the body” (page 21). Although this interpretation is supported by their findings, the authors never tested the mediation. Therefore, it might be beneficial to qualify some of these statements, as causality cannot be currently assessed.

We believe that our existing manipulations directly demonstrate this casual link. We hypothesize that essentialized traits should be seen as (a) more likely to be embodied, that is, to “show up” in the brain; (b) more likely to be innate, and (c) more serious and immutable (e.g., more severe, associated with poorer prognosis). Experiments 1 & 3 both demonstrates that this is indeed the case. As such, our results show that the type of symptom (visual or decoding) *causes* people to exhibit attitudes that are associated with essentialist reasoning. Experiments 2 & 3 further shows that this is the case for *all* symptoms perceived as embodied. We now highlight this causal link in the General Discussion (on p. 28).

2. Figures 1 and 2 did not display correctly on my screen. There was a weird ? box in several places which made it look weird and slightly challenging to read. I am assuming this was not intentional, so it might be worth looking into why it is happening.

We are sorry about that, but there are no such boxes on our end; the figures look fine on our end. To eliminate any doubts, we also provide a pdf file, where all figures show correctly.

**Reviewer #2**: This revision is a much improved version. The researchers have been very responsive with regard to reviewers' comments. The introduction is more theoretically grounded and coherent. My only question is that given the number of t-tests and ANOVAs being conducted, e.g., 7 repeated-measures ANOVAs, no multiple comparison correction was applied. What were the rationales? The p-values reported in the results may not stand the test of correction. I think it's fine given the exploratory nature of the study. It's better to include it, rather than reporting the "significant" results. Overall, I think this study is interesting, and i see the challenge of doing interdisciplinary research like this one. I think readers would be interested in learning more about how dualism and essentialism could be potentially related to public misconception of dyslexia.

Thank you for the careful read and helpful comments. Usually, correction is applied when a manipulation of interest is represented by multiple cells. In our design, however, the seven tests each test distinct questions; so conceptually, these are distinct manipulations. For this reason, we do not believe that a correction of the p-value is in order.
